# Pancreatic Steatosis in Patients with Pheochromocytoma and Paraganglioma: A Retrospective Single-Center Study

**DOI:** 10.3390/jcm15062416

**Published:** 2026-03-21

**Authors:** Belma Özlem Tural Balsak, Betül Akdal Dölek, Muzaffer Serdar Deniz, Gökhan Rıza Baykal, Narin Nasıroğlu Imga, Oya Topaloğlu, Reyhan Ersoy, Bekir Çakır

**Affiliations:** 1 Department of Endocrinology, Ankara City Hospital, Ankara 06800, Turkey; serdardeniz86@hotmail.com (M.S.D.); gokhanbaykal89@yahoo.com (G.R.B.); narinnasiroglu@gmail.com (N.N.I.); oyasude@gmail.com (O.T.); reyhanersoy@yahoo.com.tr (R.E.); drcakir@yahoo.com (B.Ç.); 2 Department of Endocrinology, University of Health Sciences, Ankara 06800, Turkey; 3 Department of Radiology, Ankara City Hospital, Ankara 06800, Turkey; b_akdal@yahoo.com; 4 Department of Endocrinology, Faculty of Medicine, Ankara Yildirim Beyazit University, Ankara 06800, Turkey

**Keywords:** catecholamines, obesity, pheochromocytoma, pancreatic steatosis, triglyceride–glucose index, computed tomography

## Abstract

**Background/Objectives**: This study aimed to evaluate pancreatic steatosis and its association with the triglyceride index in patients with pheochromocytoma and paraganglioma (PPGL). **Methods:** In this retrospective single-center study conducted between 2 January 2019 and 30 April 2024, thirty-three patients with confirmed PPGL and thirty-three age-, sex-, and body mass index (BMI)-matched healthy controls were evaluated. The mean age of the study population was 49.7 ± 12.3 years, and 32 participants (48.5%) were female. Pancreatic fat infiltration was assessed via computed tomography (CT). Body composition parameters, including visceral fat area (VFA), subcutaneous fat area (SFA), skeletal muscle area (SMA), and skeletal muscle area index (SMAI), were also measured. Laboratory data, including lipid profiles, glucose, hemoglobin A1c (HbA1c), and triglyceride index (TyG), were analyzed. **Results:** Pancreatic steatosis, as indicated by significantly lower pancreatic attenuation values, was markedly higher in the PPGL group compared to controls (*p* < 0.001). No significant differences were observed between the groups in terms of VFA (*p* = 0.218), SFA (*p* = 0.413), SMA (*p* = 0.669), or SMAI (*p* = 0.562). Pancreatic fat was positively correlated with triglyceride levels, VLDL, TyG index, BMI, and VFA. No correlation was found between catecholamine levels and pancreatic steatosis. **Conclusions**: Our findings indicate an increase in pancreatic steatosis in patients with PPGL, suggesting a potential association between catecholamine excess and the development of ectopic fat deposition. These results support considering pancreatic fat as a marker of metabolic dysfunction in PPGL.

## 1. Introduction

Pheochromocytomas and paragangliomas (PPGLs) are rare catecholamine-secreting neuroendocrine tumors arising from chromaffin cells of the adrenal medulla or extra-adrenal paraganglia [1]. Approximately 80–85% of cases are pheochromocytomas, whereas 15–20% are paragangliomas [2]. Although most cases occur sporadically, a considerable proportion is associated with hereditary syndromes involving germline mutations [3]. Imaging plays a crucial role in the localization and characterization of these tumors, and computed tomography (CT) is widely used as the initial imaging modality for tumor detection and evaluation [4].

The metabolic consequences of catecholamine excess are well documented and include hypertension, impaired glucose metabolism, insulin resistance, and secondary diabetes mellitus [5]. In addition, catecholamine-induced lipolysis may alter body composition and contribute to abnormal fat distribution. These metabolic alterations may influence ectopic fat deposition in various organs [6].

Ectopic fat accumulation in non-adipose tissues has emerged as an important marker of metabolic dysfunction. Among these, pancreatic steatosis—also referred to as fatty pancreas—has gained increasing attention in recent years [7]. Pancreatic steatosis has been associated with obesity, increased body mass index (BMI), insulin resistance, metabolic syndrome, and type 2 diabetes mellitus [8,9]. The prevalence of pancreatic steatosis has been reported to range between 30% and 33% [10]. Among metabolic parameters, triglyceride levels and HbA1c have been identified as the strongest predictors of pancreatic steatosis [11].

Various imaging modalities, including ultrasonography, computed tomography (CT), and magnetic resonance imaging (MRI), can be used to evaluate pancreatic steatosis. In CT-based assessments, pancreatic fat accumulation is commonly quantified using pancreatic attenuation values or pancreas-to-spleen attenuation ratios. Lower pancreatic attenuation values indicate increased fat deposition within the pancreatic parenchyma, making CT a useful and widely available tool for assessing pancreatic steatosis in clinical practice [12].

Although the metabolic consequences of catecholamine excess in PPGL are well established, the potential relationship between PPGL and ectopic fat accumulation remains poorly understood. In particular, data regarding pancreatic steatosis in patients with PPGL are limited, and the extent to which catecholamine excess may contribute to pancreatic fat deposition has not been well investigated.

Therefore, this study aimed to evaluate pancreatic steatosis in patients with PPGL using computed tomography and to investigate its potential association with metabolic parameters.

## 2. Materials and Methods

### 2.1. Study Design

This retrospective single-center study was conducted at the Endocrinology Clinic of Ankara City Hospital between 2 January 2019, and 30 April 2024. Adult patients (≥18 years) with a diagnosis of PPGL were included in the study. Control individuals underwent abdominal CT imaging for clinical indications unrelated to pancreatic or metabolic disease, such as evaluation of non-specific abdominal pain or preoperative assessment (e.g., inguinal hernia surgery). Basic biochemical parameters and lipid profiles were evaluated in individuals considered for the control group. Only those with normal kidney function tests and without known chronic disease or regular medication use were included. The control group was matched with the PPGL group in terms of age, sex, and body mass index (BMI).

**Figure 1:**
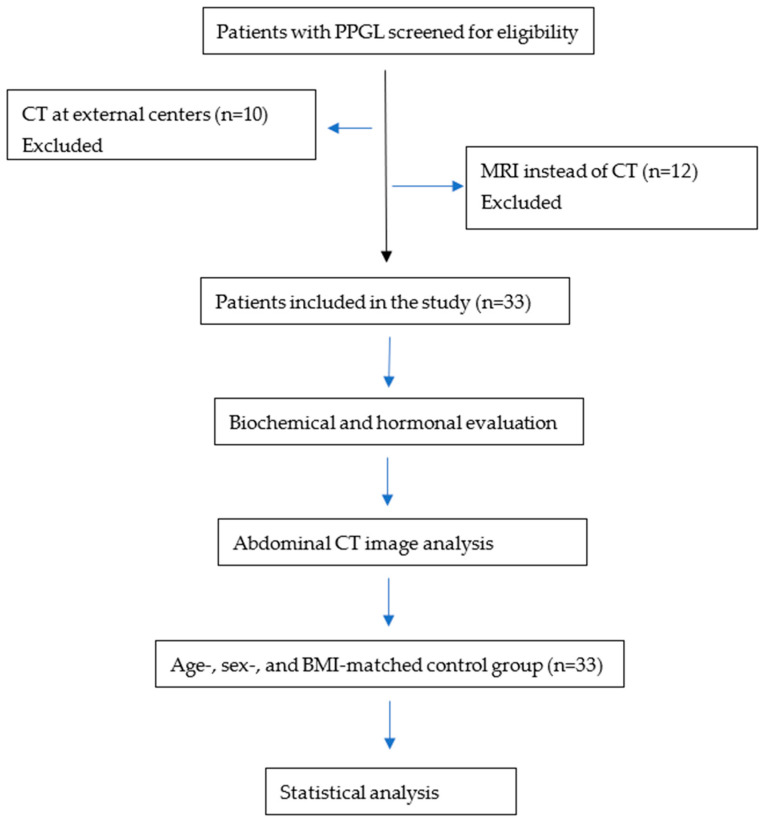
Flow diagram of patient selection.

A total of 55 patients with PPGL were initially screened for eligibility. Ten patients had CT scans performed at external institutions and were excluded because the imaging data were not obtained at our center. In addition, twelve patients were evaluated with magnetic resonance imaging (MRI) rather than CT and were therefore excluded from the study (Figure 1).

The study was approved by the Ethics Committee of Ankara City Hospital (Approval No: TABED 2-24-205).

#### 2.1.1. Inclusion and Exclusion Criteria

Inclusion criteria

- Patients aged 18 years and older.
- Patients with a confirmed diagnosis of pheochromocytoma or paraganglioma (PPGL) who were evaluated at our institution.
- Availability of abdominal computed tomography (CT) imaging for the assessment of pancreatic steatosis.
- For the control group: individuals who underwent abdominal CT for any reason at our hospital and had no history of chronic disease or regular medication use.

Exclusion criteria

- History of alcohol use, to eliminate potential confounding effects on pancreatic fat accumulation.
- Absence of abdominal CT imaging, since pancreatic steatosis was evaluated using CT in this study.

#### 2.1.2. Study Protocol

The participants’ age, gender, height, weight, BMI, biochemical variables, and catecholamine profiles were examined.

Abdominal computed tomography (CT) scans were used to evaluate pancreatic structures and body composition parameters.

The lesion sizes of PPGL were recorded. From CT images, liver size, hepatic fat attenuation (Hounsfield units, HU), lesion attenuation, visceral fat area (VFA), subcutaneous fat area (SFA), skeletal muscle area (SMA), pancreatic fat attenuation, and splenic attenuation were measured and recorded by the same experienced radiologist who was blinded to the clinical and laboratory data.

The skeletal muscle area index (SMAI) was calculated by dividing the skeletal muscle area (cm^2^) by the square of height (m^2^). Sarcopenia was defined as an SMAI of <52.4 cm^2^/m^2^ in men and <38.5 cm^2^/m^2^ in women.

Only patients whose postoperative pathology reports confirmed PPGL were included in the study. Comorbidities of the patients were recorded. Those with hypertension, diabetes, prediabetes, and hyperlipidemia were determined.

The endocrine evaluation included measurements of plasma and 24 h urinary catecholamines and metanephrines, including epinephrine, norepinephrine, metanephrine, normetanephrine, and dopamine.

The control group did not undergo endocrine testing, as these individuals had no clinical suspicion of endocrine disease and had no history of chronic illness.

The triglyceride–glucose (TyG) index, calculated as ln [fasting triglycerides (mg/dL) × fasting glucose (mg/dL)/2], has been widely accepted as a surrogate marker of insulin resistance.

#### 2.1.3. Blood and Urinary Markers

Morning fasting glucose (70–99 mg/dL), liver function tests, kidney function tests, triglycerides (TG) (<150 mg/dL), High-density Lipoprotein (HDL) (>50 mg/dL), Very Low-Density Lipoprotein (VLDL) (10–40 mg/dL), Low-Density Lipoprotein (LDL) (100–129 mg/dL), total cholesterol (<200 mg/dL) and Hemoglobin A1c (HbA1c) (<5.7%) levels were measured in all patients. Serum insulin concentrations (3–25 mU/L) were studied. Plasma glucose and liver function tests were analyzed by the spectrophotometric method with the Siemens Atellica device (Siemens Healthineers, Erlangen, Germany). Insulin was studied by the chemiluminescence (CLIA) method with the Siemens Atellica device (Siemens Healthineers, Erlangen, Germany).

Thyroid-stimulating hormone (TSH, 0.55–4.78 mU/L) levels were measured. Hormone profiles were measured in the morning with the device Siemens Atellica IM 1600 and using an immunoassay method ((Siemens Healthineers, Erlangen, Germany). Plasma adrenaline concentrations were measured using high-performance liquid chromatography (HPLC), while 24 h urinary catecholamines were analyzed by LC-MS/MS using SCIEX 5500 and 5500 PLUS systems (SCIEX, Framingham, MA, USA).

#### 2.1.4. Radiological Evaluation

All abdominal CT scans were performed using a 128-slice CT scanner (GE Revolution EVO, GE Medical Systems, Milwaukee, WI, USA) with patients in the supine position during full inspiration. The scans were acquired without intravenous contrast administration. The scanning protocol included a tube voltage of 120 kV with an automated tube current modulation system. The pitch factor was 0.98, and axial images were reconstructed with a slice thickness of 1.3 mm using a sharp convolution kernel.

#### 2.1.5. Image Analysis

CT images were analyzed to evaluate hepatic, pancreatic, adipose, and skeletal muscle attenuation.

Hepatic attenuation was measured using three regions of interest (ROIs) placed in different liver segments while avoiding vessels, bile ducts, and focal lesions. Splenic attenuation was measured using two ROIs within the splenic parenchyma. Pancreatic attenuation was measured separately in the head, body, and tail of the pancreas, carefully avoiding pancreatic ducts, vessels, and peripancreatic fat. Hepatic steatosis was defined as a spleen-to-liver attenuation difference greater than 10 Hounsfield units (HU).

Body composition parameters were evaluated at the third lumbar vertebra (L3) level using a single axial CT slice. Visceral fat area (VFA) and subcutaneous fat area (SFA) were segmented semi-automatically within a predefined attenuation range of −190 to −30 HU. Manual adjustments were performed when necessary to accurately define tissue boundaries.

The cross-sectional skeletal muscle area at the L3 level was also measured. The analyzed muscle groups included the psoas, erector spinae, quadratus lumborum, transversus abdominis, internal and external obliques, and rectus abdominis muscles. Skeletal muscle was identified using an attenuation threshold of −29 to +150 HU, and the total cross-sectional muscle area was calculated in cm^2^.

#### 2.1.6. Adrenal Lesion Evaluation

For patients with adrenal lesions, the largest axial diameter of each lesion was recorded. Attenuation measurements were obtained by placing ROIs within the most homogeneous solid portion of the lesion while avoiding areas of necrosis, calcification, or cystic change. Morphological assessment included evaluation for the presence of necrosis, calcification, and cystic components, based on visual inspection and attenuation characteristics.

#### 2.1.7. Statistical Analysis

Quantitative variables that do not comply with normal distribution are presented as median and minimum-maximum values. Normally distributed quantitative variables of the two groups were analyzed with the Student t-test. Non-parametric variables were compared with the Mann–Whitney U test. Chi-square tests were used to analyze differences between groups for nominal variables. Statistical significance was defined as a *p*-value < 0.05. Pearson correlation or Spearman correlation analyses were conducted to investigate the correlation between the quantitative variables according to the distributions.

## 3. Results

### Demographic and Clinical Characteristics

A total of 33 patients with PPGL and 33 healthy controls were included in the study. The groups were matched for age, sex, and body mass index (BMI). The mean age was comparable between the groups (49.64 ± 11.88 years in the PPGL group and 49.85 ± 12.81 years in the control group), with an age range of 19–70 years in the PPGL group and 21–75 years in the control group.

Tumor characteristics of the PPGL patients are presented in Table 1. The median tumor size was 37 mm (IQR: 21; range 21–73 mm). Lesions were located in the right adrenal gland in 14 patients, in the left adrenal gland in 13 patients, and bilaterally in 3 patients. Three patients had extra-adrenal paragangliomas. All patients underwent laparoscopic surgery.

No statistically significant differences were observed between the groups in terms of lipid profiles or triglyceride index. However, fasting glucose and HbA1c levels were statistically significantly higher in the PPGL group compared with the control group (*p* = 0.006 and *p* = 0.032, respectively) (Table 2). The age and laboratory characteristics of the study population are presented in Table 2.

**Table 2:** Comparison of age, BMI and laboratory characteristics of the groups.

	Pheochromocytoma (*n* = 33)	Control (*n* = 33)	t or U	*p*
Age (years)	49.64 ± 11.88	49.85 ± 12.81	0.070	0.945
BMI (kg/m^2^)	27.98 ± 4.88	27.24 ± 3.69	0.700	0.487
Glucose (70–99 mg/dL)	104 ± 32.26	88 ± 6.45	2.830	0.006
Creatinine (0.6–1.2 mg/dL)	0.782 ± 0.17	0.785 ± 0.18	0.069	0.945
ALT (7–56 U/L)	28.2 ± 14.5	23.8 ± 12.3	1.308	0.196
LDL-C (100–129 mg/dL)	107.9 ± 36.6	124.6 ± 31.9	1.944	0.056
TG (<150 mg/dL)	151.1 ± 82.5	141.7 ± 65	0.509	0.612
VLDL-C (10–40 mg/dL)	30.2 ± 16.5	28.3 ± 13.0	0.503	0.617
HDL-C (>50 mg/dL)	46.5 ± 13.3	43.8 ± 9.8	0.924	0.359
Total Chol (<200 mg/dL)	184 ± 42.3	197 ± 34.5	1.346	0.183
HbA1c (<5.7%)	6.057 ± 0.964	5.623 ± 0.293	2.201	0.032
TyG	8.78 ± 0.69	8.63 ± 0.49	0.985	0.329
TSH (0.55–4.78 mU/L)	1.821 ± 1.187	1.707 ± 0.926	0.413	0.681

ALT: alanine aminotransferase, BMI: body mass index, HDL-C: high-density lipoprotein, LDL-C: low-density lipoprotein, TG: triglyceride, Total Chol: total cholesterol, TSH: thyroid-stimulating hormone, TyG: triglyceride glucose index, VLDL-C: very low-density lipoprotein, There were no significant differences between the groups in terms of obesity, overweight, or sarcopenia. However, hypertension (75.8%), hyperlipidemia (45.5%), type 2 diabetes mellitus (24.2%), and coronary artery disease (15.2%) were significantly more prevalent in the PPGL group compared to the control group (*p* = 0.039, *p* < 0.001, *p* = 0.003, and *p* = 0.020, respectively). The prevalence of prediabetes was 15.2% in the PPGL group and 30.2% in the control group, which was not statistically significant (*p* = 0.142) (Table 3).

**Table 3:** Sex distribution and comorbidities in the groups.

	Pheochromocytoma (*n* = 33)	Control (*n* = 33)	X^2^	*p*
Sex (female *n* (%))	16 (48.5)	16 (48.5)	0.000	1.000
Sarcopenia (*n* (%))	5 (15.2)	4 (12.1)	0.129	0.720
Obesity (*n* (%))	10 (30.3)	10 (30.3)	0.000	1.000
Overweight (*n* (%))	13(39.4)	14 (42.4)	0.063	0.802
Hyperlipidemia (*n* (%))	15 (45.5)	0 (0)	19.412	0.039
Hypertension (*n* (%))	25 (75.8)	0 (0)	40.244	0.000
Coronary heart disease (*n* (%))	5 (15.2)	0 (0)	5.410	0.020
Type 2 Diabetes (*n* (%))	8 (24.2)	0 (0)	9.103	0.003
Prediabetes (*n* (%))	5 (15.2)	10 (30.3)	2.157	0.142

Pancreatic fat infiltration was significantly higher in patients with PPGL (*p* < 0.001). In addition, liver size and liver HU were significantly higher in the PPGL group (*p* = 0.015 and *p* = 0.006, respectively). There was no significant difference between the groups in terms of hepatic steatosis (*p* = 0.738). Likewise, VFA, SFA, SMA, and SMAI values were comparable between the groups. Comparison of body composition measurements between PPGL and controls is presented in Table 4.

Correlations between body composition parameters and catecholamine levels were examined (Table 5). VFA was found to be positively correlated with plasma normetanephrine and urinary normetanephrine levels. The SMAI was associated with plasma adrenaline levels. Tumor size showed a significant correlation with plasma metanephrine, plasma normetanephrine, 24 h urinary metanephrine, and 24 h urinary normetanephrine levels.

Pancreatic attenuation was found to be correlated with triglyceride levels, VLDL, triglyceride index, BMI, and VFA. No significant association was observed between liver size and lipid parameters (Table 6).

## 4. Discussion

This study examined pancreatic steatosis and its relationship with insulin resistance in PPGL patients.

The initial finding revealed a significantly higher prevalence of pancreatic steatosis in the PPGL group. The second finding was that patients with PPGL had significantly higher levels of glucose, HbA1c, and triglyceride index compared to the control group. Pancreatic steatosis, traditionally linked to obesity, insulin resistance, and metabolic syndrome, has recently gained attention as a marker of systemic metabolic dysfunction. Pancreatic steatosis itself has been associated with inflammatory and fibrotic changes that may impair both exocrine and endocrine pancreatic functions [13]. The triglyceride–glucose (TyG) index is widely used as a surrogate marker for insulin resistance [14]. It has been demonstrated that the TyG index reflects insulin resistance to a similar extent as the hyperinsulinemic–euglycemic clamp test [15]. In a study conducted by Andrade et al., a positive correlation was found between the TyG index and the degree of pancreatic steatosis. The authors reported that the TyG index performed better than HOMA-IR in reflecting this association [16].

In the study conducted by Okamura et al., glucose intolerance was more frequently observed in the pheochromocytoma group compared to controls (51% vs. 17) [17]. Similarly, in our study, glucose and HbA1c levels were significantly higher in the pheochromocytoma group.

In the literature, the prevalence of diabetes in patients with pheochromocytoma has been reported to range between 21% and 37% [18]. The findings of our study, showing a 24.2% prevalence of type 2 diabetes, are consistent with these previously reported rates. Both impaired insulin secretion and increased insulin resistance are considered contributing factors in the pathogenesis of diabetes in pheochromocytoma [19,20]. Catecholamines reduce insulin secretion by exerting an inhibitory effect on pancreatic β-cells, primarily through stimulation of α2-adrenergic receptors [21,22]. The pathophysiological basis for insulin resistance in PPGL is thought to involve catecholamine-induced hepatic glucose overproduction and reduced peripheral glucose uptake [23]. Additionally, elevated levels of free fatty acids, along with decreased levels of adiponectin and leptin, are also considered contributing factors to increased insulin resistance [24,25,26]. In addition to all these factors, pancreatic steatosis may represent an additional mechanism contributing to insulin resistance. However, the impairment of beta-cell function due to fat infiltration may take many years to manifest [27].

Therefore, while catecholamine excess appears to play a role in metabolic dysregulation, the mechanisms underlying insulin resistance in PPGL require further elucidation.

The third finding of this study is that pancreatic steatosis was positively correlated with triglyceride levels, VLDL, and BMI. Xiao et al. evaluated 1774 healthy individuals (928 females and 846 males) for pancreatic steatosis using ultrasound. Among them, 44.94% of females and 50.0% of males were diagnosed with non-alcoholic fatty pancreas disease. Pancreatic fat accumulation was found to be positively associated with age, weight, BMI, TyG index, TyG-BMI, LDL, total cholesterol, TG, fasting plasma glucose, TG/HDL ratio, and uric acid levels. Conversely, a negative correlation was observed with HDL levels. Their findings indicate that parameters related to body weight and triglycerides are significant predictors of NAFPD, with BMI emerging as the most powerful indicator [28]. In our study, although there were no significant differences between the PPGL and control groups in terms of triglyceride levels, VLDL, or BMI, pancreatic steatosis was positively correlated with these variables. This finding suggests that PPGL may play a role in the development of metabolic disturbances.

The fourth finding is that pancreatic steatosis was found to be positively correlated with VFA in our study. Liver size was found to be larger in the pheochromocytoma group; however, liver attenuation values (HU) were also higher, indicating a lower degree of hepatic steatosis. There was no significant difference between the groups in terms of hepatic steatosis. Although no significant difference in VFA was observed between the patient and control groups overall, among PPGL patients, those with pancreatic steatosis had significantly higher VFA values. In a study by Jaghutriz et al., pancreatic steatosis was evaluated using MRI in prediabetic individuals, and it was found to be positively associated with BMI, total body fat, and visceral fat accumulation [29]. In their study, no association was found between hepatic steatosis and pancreatic steatosis. They suggested that pancreatic fat may independently contribute to insulin resistance and the development of type 2 diabetes.

Okamura et al. compared 42 patients with pheochromocytoma and 23 with non-functioning adrenal adenomas (NFA), reporting significantly lower VFA and SFA in the pheochromocytoma group. Moreover, both parameters increased following surgical tumor removal, suggesting a strong lipolytic effect of catecholamines [17]. However, their study did not assess pancreatic fat. Importantly, their sample consisted of patients with normal BMI (Pheochromocytoma: 22.2 ± 2.9; NFA: 23.2 ± 2.4). In contrast, the majority of our patients were either overweight or obese, with BMI values comparable between the pheochromocytoma and control groups. This similarity in body composition may partly explain the absence of significant differences in fat distribution in our groups. Furthermore, previous research has demonstrated that the lipolytic response to catecholamines is attenuated in individuals with obesity, which may have obscured the expected effects on adipose tissue [30]. Specifically, a reduction in β_2_-adrenoceptor expression in upper-body adipose tissue has been associated with catecholamine resistance [31].

Ko et al. evaluated body composition changes in 313 patients with pheochromocytoma using CT before and after surgical resection. They reported a significant postoperative increase in VFA by 14.5% and SFA by 15.8%, whereas no significant changes were observed in SMA or SMAI. Visceral obesity increased following tumor removal, yet sarcopenia status remained unchanged. Interestingly, they found that higher preoperative catecholamine levels were associated with a greater postoperative increase in subcutaneous fat, suggesting a dose-dependent lipolytic effect. While sympathetic overactivity appears to enhance lipolysis in both visceral and subcutaneous adipose tissue, its direct impact on skeletal muscle remains unclear. It is important to note that the study population had a mean BMI of less than 25 kg/m^2^ [32]. Moreover, the absence of a control group and the exclusive inclusion of Korean patients may limit the generalizability of the findings to other ethnic populations.

Importantly, our cohort included a high proportion of overweight and obese individuals in both groups, which may have blunted the lipolytic effects of catecholamines due to reduced β-adrenergic sensitivity in adipose tissue, as previously reported. Therefore, while Ko et al.’s findings support a strong catecholamine-mediated lipolytic effect, our results highlight the complex interplay between obesity, sympathetic activity, and fat distribution in PPGL patients.

The fifth finding of this study is that the tumor size was found to be positively correlated with plasma metanephrine, plasma normetanephrine, urinary metanephrine, and urinary normetanephrine levels. Despite the significant elevation in catecholamines in PPGL, we did not observe a direct correlation between catecholamine levels and pancreatic fat. Okamura et al. reported a correlation between tumor size and urinary normetanephrine levels. This may be due to individual variability in catecholamine receptor sensitivity, baseline metabolic status, or differences in tissue-specific lipid handling.

This study has several limitations, including its retrospective design and relatively small sample size. Although the control group was matched for age, sex, and BMI, the lack of fully standardized evaluation of the control group may introduce potential selection bias. Furthermore, insulin measurements were not available for all patients; therefore, the triglyceride–glucose (TyG) index was used as a surrogate marker of insulin resistance. Finally, the absence of longitudinal data limits our ability to assess changes over time. Prospective evaluations before and after surgery would provide more comprehensive insight into catecholamine-related fat redistribution.

Despite these limitations, our study contributes to a growing body of literature emphasizing the metabolic implications of catecholamine excess. Clinicians should be aware of the potential for pancreatic fat accumulation and related metabolic disturbances in patients with pheochromocytoma.

## 5. Conclusions

Herein we demonstrated that pancreatic steatosis was significantly more prevalent in patients with PPGL compared to controls. Although no significant differences were observed between the groups in terms of BMI, triglyceride levels, or VLDL, pancreatic fat showed a positive correlation with these metabolic parameters, supporting the presence of metabolic dysfunction in the PPGL group. Additionally, patients with PPGL had higher glucose, HbA1c, and triglyceride index levels, further suggesting a link between catecholamine excess and metabolic alterations. Although hepatic steatosis did not differ significantly between the groups, and liver attenuation values were higher in the PPGL group, pancreatic steatosis was notably associated with components of the metabolic syndrome. These results support the consideration of pancreatic fat as a novel and clinically meaningful marker of metabolic dysfunction in PPGL patients.

## Figures and Tables

**Table 1:** Tumor characteristics of patients with PPGL.

Variable	PPGL Patients (*n* = 33)
Tumor size, median (min–max), mm	37 (21–73)
Right adrenal gland	14 (42.4%)
Left adrenal gland	13 (39.4%)
Bilateral adrenal gland	3 (9.1%)
Extra-adrenal paraganglioma	3 (9.1%)
Laparoscopic surgery	33 (100%)

**Table 4:** Comparison of body composition measurements between pheochromocytoma patients and controls.

	Pheochromocytoma (*n* = 33)	Control (*n* = 33)	t or U	*p*
Liver size	166.14 ± 19.90	154.30 ± 17.94	2.487	0.015
VFA (M (IQR))	161.40 (146.82)	145.81 (92.61)	1.515	0.218
SFA	215.11 ± 87.89	233.11 ± 32.20	−0.823	0.413
SMA	154.51 ± 36.69	150.86 ± 32.20	0.429	0.669
Liver Fat HU (M (IQR))	60 (7)	56 (10)	7.570	0.006
Pancreas Fat HU	34.71 ± 11.01	42.39 ± 3.93	−3.761	<0.001
Spleen HU	47.87 ± 3.07	48 ± 5.87	−0.109	0.914
SMAI	53.65 ± 10.16	52.29 ± 8.86	0.538	0.562

HU: Hounsfield unit, SFA: subcutaneous fat area, SMA: Smooth muscle area, SMAI: Smooth muscle area index, VFA: visceral fat area, M: Median, IQR: Interquartile Range.

**Table 5:** The relationship between catecholamines and body composition measurements in patients with pheochromocytoma.

	PMET (r; *p*)	PNMET	PADR	UMET	UMET Ratio	UNMET
Liver size	0.283 0.117	0.175 0.337	0.170 0.616	0.253 0.156	0.224 0.209	0.069 0.703
Pancreas fat HU	0.322 0.083	0.348 0.060	0.128 0.708	0.260 0.158	0.297 0.105	0.034 0.854
VFA	−0.262 0.148	0.351 0.049	−0.289 0.388	−0.199 0.266	−0.190 0.290	−0.449 0.009
SFA	−0.066 0.721	0.040 0.828	−0.145 0.670	−0.056 0.759	−0.020 0.910	0.133 0.460
SMA	−0.296 0.100	−0.005 0.978	−0.374 0.257	−0.259 0.145	−0.239 0.180	−0.012 0.947
SMAI	0.326 0.069	0.041 0.824	0.687 0.019	0.252 0.157	0.241 0.177	0.027 0.879
Spleen HU	0.010 0.960	−0.278 0.137	0.489 0.127	0.008 0.968	0.030 0.873	−0.221 0.233
Liver HU	0.219 0.244	−0.165 0.383	0.179 0.599	0.267 0.146	0.217 0.240	0.010 0.958
Lesion size	0.389 0.028	0.519 0.003	0.20 0.953	0.340 0.053	0.362 0.039	0.380 0.029

SFA: subcutaneous fat area, SMA: smooth muscle area, SMAI: smooth muscle area index, VFA: visceral fat area, PMET: plasma metanephrine, PNMET: plasma normetanephrine, PADR: plasma adrenaline, UMET: urinary metanephrine, UMET ratio: urinary metanephrine to creatinine ratio, UNMET: urinary normetanephrine, HU: Hounsfield unit.

**Table 6:** The Correlations Between Body Composition Measurements and Lipid Profiles in Patients with Pheochromocytoma.

	LDL	TG	VLDL	HDL	T. CHOL	BMI	TyG Index	Liver Size	Pancreas Fat HU	Liver HU	VFA	SFA
Liver size	0.096 0.608	0.186 0.318	0.187 0.313	0.126 0.500	0.046 0.804	0.039 0.757	0.232 0.065	1	−0.017 0.896	0.180 0.155	−0.135 0.278	0.089 0.476
Pancreas fat HU	−0.170 0.379	−0.465 0.011	−0.455 0.013	0.202 0.293	−0.322 0.088	−0.589 0.000	−0.528 0.003	−0.017 0.896	1	0.186 0.141	0.690 0.000	0.257 0.162
VFA	0.096 0.606	0.505 0.004	0.505 0.004	−0.371 0.040	0.161 0.387	0.679 0.000	0.472 0.007	−0.135 0.278	0.690 0.000	−0.318 0.081	1	0.381 0.029
SFA	0.141 0.451	0.202 0.275	0.208 0.262	0.167 0.369	0.246 0.182	0.676 0.000	0.248 0.179	0.089 0.476	0.257 0.162	−0.0.59 0.646	0.381 0.029	1
SMA	−0.085 0.651	0.238 0.198	0.235 0.203	−0.388 0.031	−0.116 0.535	0.195 0.276	0.146 0.433	−0.260 0.035	0.174 0.350	−0.251 0.045	0.415 0.016	0.194 0.279
SMAI	0.000 0.999	0.200 0.282	0.197 0.288	−0.257 0.040	0.041 0.828	0.285 0.020	0.152 0.414	−0.255 0.039	0.268 0.144	−0.243 0.053	0.503 0.003	0.009 0.961
Lesion size	−0.012 0.947	−0.094 0.617	−0.097 0.603	0.029 0.875	−0.030 0.874	−0.319 0.070	−0.113 0.546	−0.286 0.106	−0.055 0.770	0.088 0.636	−0.139 0.439	−0.238 0.183
TyG index	0.150 0.238	0.960 0.000	0.960 0.000	−0.423 0.000	0.327 0.008	0.306 0.014	1	0.232 0.065	0.528 0.003	0.130 0.501	0.472 0.007	0.248 0.179
BMI	0.122 0.339	0.218 0.084	0.214 0.089	0.074 0.559	0.205 0.105	1	0.306 0.014	0.039 0.757	0.589 0.000	0.228 0.216	0.679 0.000	0.676 0.000

BMI: body mass index; HDL-C: high-density lipoprotein; SFA: subcutaneous fat area; SMA: skeletal muscle area; SMAI: skeletal muscle area index; VFA: visceral fat area; HU: Hounsfield unit; LDL-C: low-density lipoprotein; TG: triglyceride; total Chol: total cholesterol; TyG: triglyceride–glucose index; VLDL-C: very low-density lipoprotein.

## Data Availability

The datasets generated and analyzed in the present study are available upon reasonable request to the corresponding author.
